# Stress-induced galectin-1 influences immune tolerance in the spleen and thymus by modulating CD45 immunoreactive lymphocytes

**DOI:** 10.1007/s12576-016-0478-8

**Published:** 2016-08-29

**Authors:** Kenichi Sasaguri, Kentaro Yamada, Yuri Narimatsu, Masami Oonuki, Azusa Oishi, Koyo Koda, Kin-ya Kubo, Toshiharu Yamamoto, Toshihiko Kadoya

**Affiliations:** 1 Department of Dentistry, Oral and Maxillofacial Surgery, Jichi Medical University, 3311-1 Yakushiji, Shimotsuke, Tochigi 329-0498 Japan; 2 Brain Functions and Neuroscience Division, Department of Oral Science, Kanagawa Dental University Graduate School, Inaoka-cho 82, Yokosuka, Kanagawa 238-0003 Japan; 3 Division of Orthodontics, Department of Oral Science, Kanagawa Dental University Graduate School, 82 Inaoka-cho, Yokosuka, Kanagawa 238-8580 Japan; 4 Department of Biotechnology, Maebashi Institute of Technology, 460-1 Kamisadori-machi, Maebashi, Gunma 371-0816 Japan; 5 Seijoh University Graduate School of Health Care Studies, 2-172 Fukinodai, Tokai, Aichi 476-8588 Japan

**Keywords:** Galectin-1, Stress, Thymus, Spleen, Apoptosis

## Abstract

Galectin-1 (Gal-1) is differentially expressed in normal and pathological tissues and regulates immune cell homeostasis. Restraint stress increases serum Gal-1 in rats. However, the function of stress-induced Gal-1 in serum is unknown. We determined if stress-induced Gal-1 in serum accumulates in immunocompetent organs as protection from physiological and/or psychological stress. Western blotting showed that the intensity of Gal-1 bands in stressed groups was significantly higher than that in controls. RT–PCR analysis indicated that the Gal-1 mRNA level did not increase after restraint stress. The numbers of Gal-1 immunoreactive cells in the splenic periarterial lymphatic sheath (PLS) and the thymus medulla of the stressed group were increased compared with those in controls. Furthermore, stress-induced Gal-1 immunoreactive cells corresponded to CD45 immunoreactive lymphocytes (CD45^+^) in the PLS of the spleen and the medulla of the thymus. Thus, stress-induced Gal-1 immediately accumulates in the spleen and thymus, and may modulate the immune response through apoptosis by binding to CD45^+^ lymphocytes in immune organs following physiological and/or psychological stress.

## Introduction

Gal-1, the first protein in the galectin family of β-galactoside-binding proteins to be discovered, is a homodimer of 14.5-kDa subunits. This protein is widely expressed in various normal and pathological tissues and organs such as skeletal muscle, heart, liver, brain, lung, thymus, spleen, and lymph nodes [1–4]. Furthermore, Gal-1 is a multifunctional protein that is implicated in a variety of biological activities [5, 6], such as immune cell homeostasis, tumorigenesis, nerve regeneration after injury, and the inflammatory response [7–12]. In particular, previous studies have demonstrated that Gal-1 induces apoptosis of activated T cells by binding the glycoprotein receptors CD7, CD43, and CD45 on the cell surface [13–15]. Thus, Gal-1 may play an important role in central and peripheral immune responses.

Our previous study first demonstrated that restraint stress markedly and rapidly induces an increase in the level of Gal-1 in the serum [16]. Furthermore, the stress-induced increase in Gal-1 in serum was prevented by destroying noradrenergic nerve terminals by pre-treatment with the neurotoxin 6-hydroxydopamine, but not bilateral adrenalectomy. Thus, stress-induced Gal-1 in serum is modulated by the sympathetic nervous system, but not the hypothalamic–pituitary–adrenal axis (HPA axis) [16]. These results suggested that the level of Gal-1 in the serum may play an important role in preventing physiological and/or psychological stress through the sympathetic nervous system. However, the function and the target organs of stress-induced Gal-1 in serum are unknown.

The purpose of this study was to investigate the localization of stress-induced Gal-1 in the spleen and thymus. Furthermore, we examined the interaction between CD45 immunoreactive (CD45^+^) lymphocytes and stress-induced Gal-1 in the thymus and spleen.

## Materials and methods

### Animals

Forty-seven male Sprague–Dawley rats (SLC Japan, Hamamatsu, Japan) ranging in age from 10 to 12 weeks (body weight 432 ± 30 g) were used. Animals were maintained in a temperature-controlled room (22 ± 3 °C) with a 12-h light/dark cycle (lights on at 7:00 a.m.). Three to five rats were housed per cage (260 mm wide, 380 mm deep, and 180 mm high) with free access to water and food. All experiments were carried out according to the Guidelines for Animal Experimentation of Kanagawa Dental University in strict compliance with the National Institutes of Health Guide for the Care and Use of Laboratory Animals.

### Stress procedures

The animals were divided into four groups, the control group (C), 30-min restraint stress group (S), 30-min restraint stress and 30-min rest group (SR30), and 30-min restraint stress and 60-min rest group (SR60). Animals were tied to a wooden board for 30 min for restraint stress. Leg fasteners held the rats in a spread-eagle supine position. For the rest period, animals were returned to their cages. We performance all stress inducement between 11:00 and 14:00.

### Sample preparations

Following decapitation under inhalational anesthesia with 2-bromo-2-chloro-1,1,1-trifluoroethane (Takeda Chemical Industries, Osaka, Japan), the thymus and spleen were harvested from each group for Western blotting and RT–PCR. These organs were collected in plastic tubes and stored at −80 °C until analysis. Some of the sample were fixed for immunohistochemistry and immunofluorescence staining. Rats that did not undergo a surgical procedure underwent a 30-min stress period, and then each organ was harvested at 30 min and 60 min after the end of the stress period. The rats in the SR30 and SR60 groups were returned to their cages at the end of the restraint period, where they had free access to food and water. We sampled all organs between 11:30 and 15:30.

### Western-blot analysis

The organs were homogenized in a fourfold volume of PBS containing 10 mM lactose and protease inhibitors (Proteo Guard EDTA-Free Protease Inhibitor Cocktail; Clontech Lab, Mountain View, CA, USA) using a Potter–Elehjem tissue homogenizer, and then the supernatants were obtained by centrifugation at 20,000 × *g* for 25 min. The protein concentration of the samples was measured using a Micro BCA Protein Assay Kit (Thermo Scientific, Rockford, IL, USA). Equal amounts of protein from each group were separated with SDS-PAGE and transferred to a polyvinylidene difluoride membrane using a semi-dry blotting apparatus (Galileo Bioscience, Cambridge, MA, USA). The transferred membranes were blocked with 10 % Block Ace (non-fat skim milk; DS Pharma Biomedical, Osaka, Japan) in Tris-buffered saline (pH 7.5) containing 0.1 % Tween 20 (TTBS) for 1 h at room temperature. Then, the membranes were incubated in rabbit anti-Gal-1 polyclonal antibodies (0.5 µg/ml) diluted in TTBS containing 10 % Block Ace for 1 h. The membranes were also incubated for 1 h in rabbit anti-glyceraldehyde 3-phosphate dehydrogenase (GAPDH) polyclonal antibodies (0.5 µg/ml; GeneTex, Irvine, CA, USA) diluted in TTBS containing 10 % Block Ace. The membranes were rinsed with TTBS and incubated with biotinylated goat anti-rabbit IgG (Dako, Glostrup, Denmark) diluted in TTBS containing 10 % Block Ace for 1 h. Then, the membrane was rinsed with TTBS and incubated with horseradish peroxidase-conjugated streptavidin (Dako) diluted in TTBS containing 10 % Block Ace for 1 h. The immunocomplexes on the membrane were visualized with chemiluminescence using Pierce Western Blotting Substrate Plus (Thermo Scientific) and detected with the LAS-4000 imaging system (GE Healthcare, Uppsala, Sweden). The amount of the detected protein was measured using ImageQuant TL (GE Healthcare).

### RNA isolation and real-time PCR

Total RNA was prepared from the thymus and spleen at C, S and SR30 using ISOGEN Reagent (Nippon Gene Co. Ltd., Toyama, Japan) according to the manufacturer’s instructions. RNA quality was judged from the ribosomal RNA pattern after electrophoresis on a 1.5 % agarose gel containing ethidium bromide and visualization with UV illumination. RNA concentrations were determined using a Bio Spec-nano spectrophotometer (Shimazu Access Corp., Yokohama, Japan). Complementary DNA (cDNA) was synthesized from total RNA with a First-strand cDNA synthesis Kit (Roche Diagnostics Ltd., Lewes, UK).

Real-time PCR was performed using the Light Cycler system (Roche). The sequences of the primers used to amplify rGal-1 were 5′-CAG GAA TCT CTT CGC TTC AAT C-3′ (forward) and 5′-CTC CCC GAA CTT TGA GAC A-3′ (reverse; PCR product: 89 bp), which were designed and synthesized by Nippon Gene Research Laboratory (Toyama, Japan). PCR amplification of Gal-1 was performed as follows: 95 °C for 10 min, followed by 40 cycles of 95 °C for 10 s, 60 °C for 15 s, and 72 °C for 15 s. Rat β-actin was used as a housekeeping control and was amplified using Light Cycler Primer sets (Search-LC, Heidelberg, Germany; 95 °C for 10 min followed by 40 cycles of 95 °C for 10 s, 60 °C for 10 s, and 72 °C for 10 s). Gene expression was reported as the ratio of the mRNA copy number of the target gene to that of β-actin for each sample.

### Immunohistochemistry and immunofluorescence

Immunohistochemistry was mainly performed according to our routine method [17]. Briefly, 16 animals were deeply anesthetized with 2-bromo-2-chloro-1,1,1-trifluoroethane (Takeda Chemical Industries). They were perfused with 0.9 % NaCl and then with 4 % paraformaldehyde and 0.2 % picric acid in 0.1 M sodium phosphate buffer (PB, pH 6.9). The thymus and spleen were rapidly dissected out and further fixed for 1 or 2 days at 4 °C. After washing in PB and immersing in 20 % sucrose, the samples were cut into 20-μm thick sagittal and transverse sections with a cryostat (HM505E; Microm, Walldorf, Germany) and thaw-mounted on gelatin-coated glass slides. The sections were washed overnight in 0.1 M PB (pH 7.4) containing 0.9 % saline (PBS) and incubated with rabbit anti-human Gal-1 antibody diluted into 0.5 μg/ml in PBS containing 1 % bovine serum albumin for 24 h at 4 °C. The antibody specificity has been reported elsewhere [16]. After washing in PBS, the sections were then incubated with biotinylated goat anti-rabbit IgG (BA-1000; Vector Laboratories, Burlingame, CA) diluted 1:100 in PBS for 1 h at room temperature. The sections were washed again in PBS and incubated with avidin–biotin–horseradish peroxidase complex (Vector Laboratories) diluted 1:200 in PBS-BSAT for 30 min at room temperature. After a final wash in PBS, the sections were reacted with 0.02 % 3,3’-diaminobenzidine tetrahydrochloride and 0.005 % hydrogen peroxide in 0.05 M Tris–HCl buffer solution (pH 7.4). Sections were then counterstained with thionin and coverslipped using Malinol (Muto Pure Chemicals, Tokyo, Japan). The numbers of Gal-1 immunoreactive cells were counted in splenic PLA, germinal center, and red pulp using printed photographs (original magnification: objective lens, ×10 and ocular lens, ×10). To identify the Gal-1 immunoreactive cell types, some sections were double stained with anti-Gal-1 (1:1000) and anti-CD45 (1:10, BD Pharmingen™, San Diego, CA, USA) after the sections were treated with Sudan Black to reduce autofluorescence [18]. Gal-1 immunoreactivity was visualized with Alexa Fluor 555-labeled donkey anti-rabbit IgG (1:200, Abcam Co., Cambridge, UK), and CD45 was visualized with Alexa Fluor 488-labeled donkey anti-mouse IgG (1:200, Abcam Co., Cambridge, UK).

### Data analysis

The intensity of Gal-1 bands on Western blots and Gal-1 mRNA expression were analyzed with multiple comparisons in three groups using the Kruskal–Wallis test. If the difference was significant, the Mann–Whitney *U* test was used in each of the two groups. Gal-1 protein and Gal-1 mRNA expression were described as the relative intensity of Gal-1 to GAPDH and the relative copy number ratio of Gal-1 to β-actin mRNA for each sample, respectively. Numbers of Gal-1 immunoreactive cells were statistically analyzed with the Tukey–Kramer test. *p* values <0.05 were considered statistically significant.

## Results

### The Gal-1 protein level, but not gene expression, was increased in the thymus and spleen after restraint stress

**Fig. 1:**
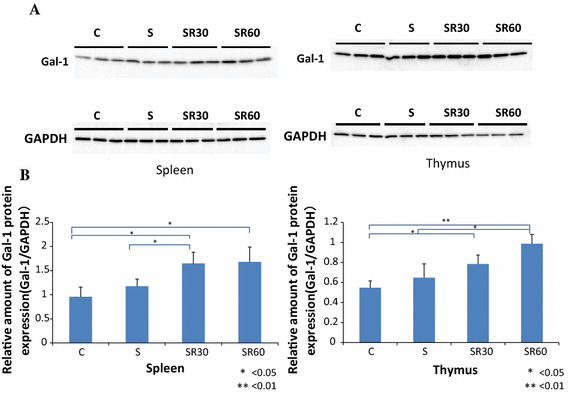
Western blotting analysis and quantitative densitometric analysis of Gal-1 protein in spleen and thymus. The spleen and thymus extracts of the C, S, SR30, and SR60 groups were subjected to western blotting analysis for Gal-1 and GAPDH. Western blotting of the spleen and thymus showed Gal-1 immunoreactive bands (14.5 kDa) and GAPDH immunoreactive bands (**a**). The amount of Gal-1 protein determined by western blotting was normalized to that of GAPDH, which was used as an internal control. The relative amount of Gal-1 is shown in the *bar graph* as the mean ± SD (**b**). *p < 0.05, **p < 0.01 (*n* = 3 rats in each group)

**Fig. 2:**
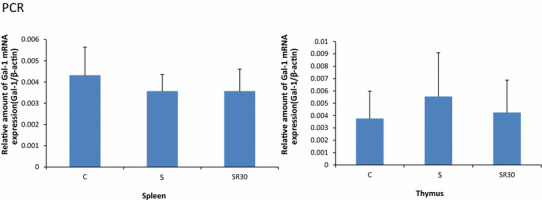
Real-time PCR analysis of Gal-1 mRNA in the thymus and spleen. The amount of galectin-1 mRNA from C, S, and SR30 rats was determined with RT–PCR and normalized to that of β-actin mRNA. Data are representative of five to seven separate determinations and are the mean ± SD. Statistical comparisons were performed for the three groups. (*n* = 5 rats in the control, and *n* = 7 each in the S and SR30 groups)

Gal-1 protein levels in the thymus and spleen were analyzed with western blotting in the C, S, SR30, and SR60 groups. The intensity of the Gal-1 bands in the SR30 and SR60 groups was stronger than in the C group in the thymus and spleen (Fig. 1a). The amount of Gal-1 protein determined with the image analyzer was normalized to that of GAPDH. Quantification showed that the increased Gal-1 levels in the thymus and spleen in the SR30 and SR60 groups were significantly higher than those in the C group (Fig. 1b). Furthermore, we examined whether increased Gal-1 protein in each organ was derived from thymocytes and splenocytes using RT–PCR to amplify the Gal-1 gene. Melting curve analysis demonstrated the presence of a single fluorescence peak representing Gal-1 mRNA, and a single band was observed with agarose gel electrophoresis in all samples (data not shown). No significant differences were observed among the three groups for Gal-1 mRNA expression at any time point (Fig. 2).

### Immunohistochemical analysis of Gal-1 in the thymus and spleen

**Fig. 3:**
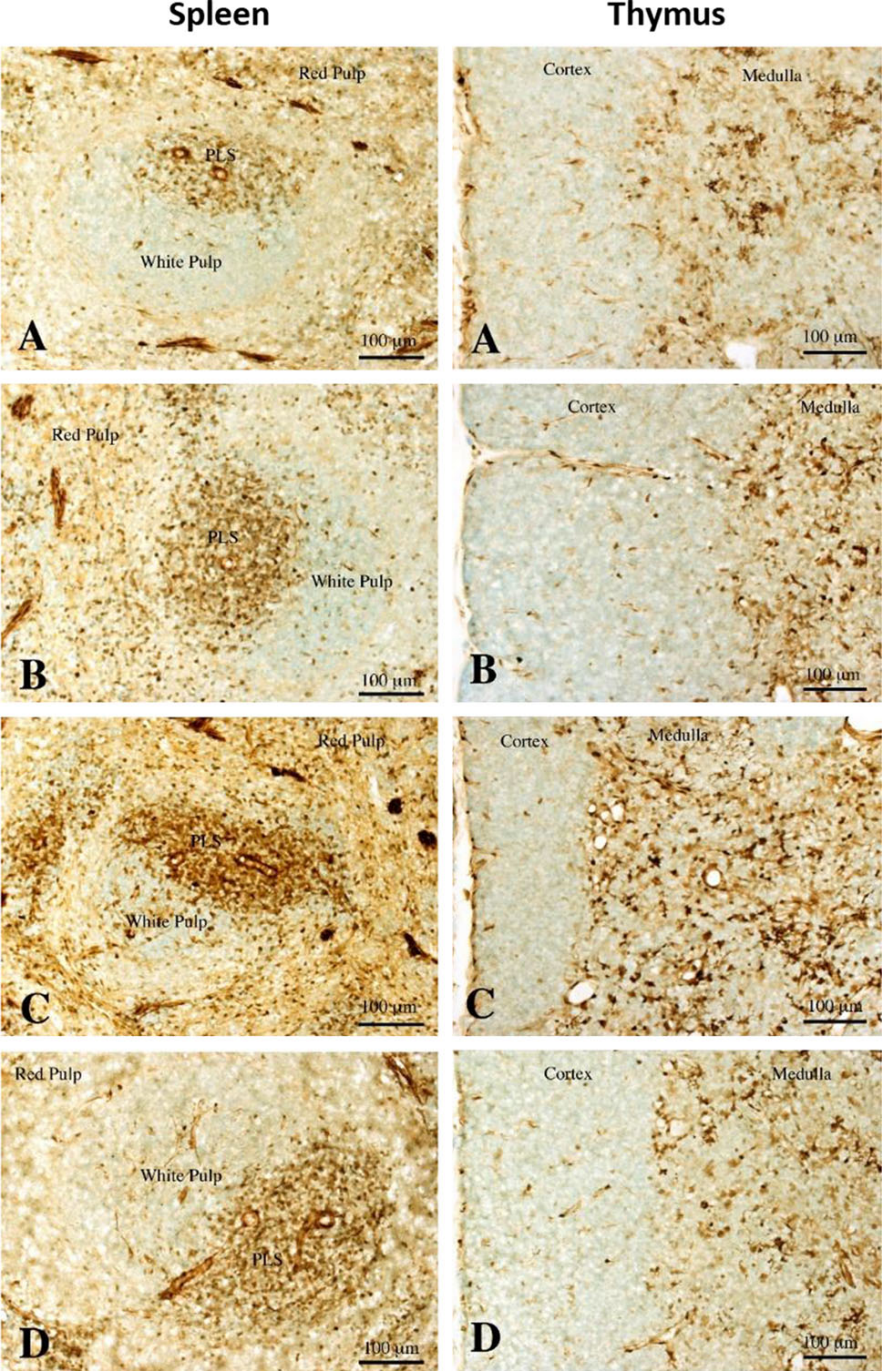
Gal-1 protein localization in the thymus and spleen of stressed rats. Change of Gal-1 immunoreactive cells in the spleen and thymus of control (**a**), just after stress (**b**), 30 min(**c**) and 60 min (**d**) survived animals after stress. Note that Gal-1 immunoreactive cells are increased in the periarterial lymphatic sheath (PLS), especially at 30 min survival animals. Gal-1 immunoreactive cells are also increased in the medulla at 30 min survival animals (**c**). *Scale bars* = 100 μm

**Fig. 4:**
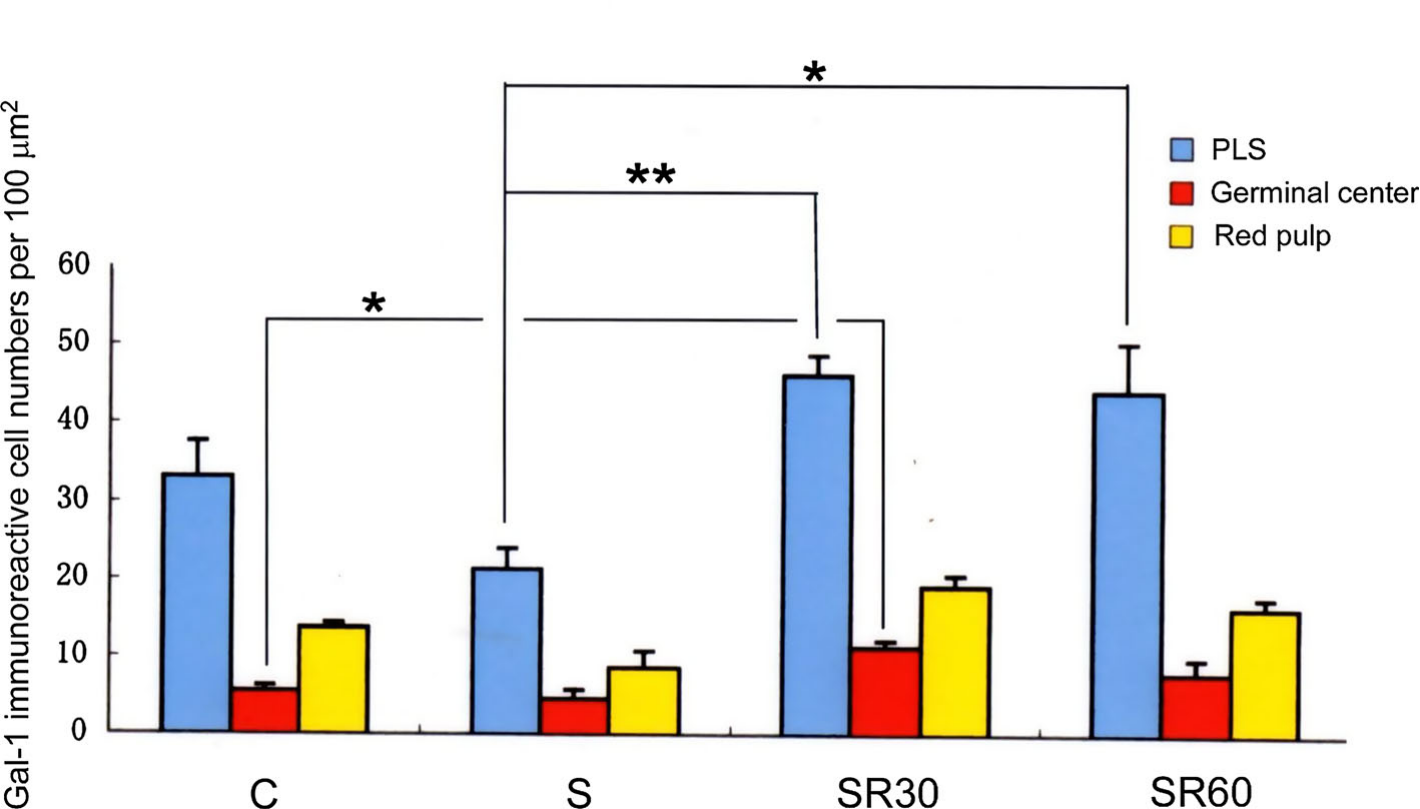
Gal-1 immunoreactive cell numbers per 100 μm^2^ in the spleen. Immunoreactive cell numbers in the periarterial lymphatic sheath (PLS), germinal center and red pulp of control (C), just after stress (S), 30-min (SR30) and 60-min (SR60) survived spleens. Note that immunoreactive cell numbers in the PLS of SR30 and SR60 are increased compared with that of the control PLS although the differences are not statistically significant. Immunoreactive cell numbers in germinal center are evidently smaller than those in the PLS, however, the cell numbers of SR30 in this region are increased compared with the control group with statistical significance. *p < 0.05, **p < 0.01 (*n* = 4 rats in each group)

We examined Gal-1 immunoreactivity in the spleen and thymus (Fig. 3). In the both organs, Gal-1 immunoreactivity was seen in lymphocytes and blood vessels (Fig. 3). The immunohistochemical analysis indicated that Gal-1-positive cells in the PLS were more densely distributed than in red pulp or germinal center in the spleen. The numbers of immunoreactive cells in the PLS and germinal center were increased after restraint stress (Fig. 4), and the PLS in SR30 and SR60 showed more abundant immunoreactive cells compared with the C and S groups (Fig. 4). In the thymus, the medulla showed a higher density of Gal-1 immunoreactive cells compared with the cortex. The intensity in the medulla increased after restraint stress, and similar to the result of the PLS in the spleen, the medulla in SR30 exhibited the densest immunoreactive cells (Fig. 3).

### Gal-1 protein and CD45^+^ lymphocytes were co-localized in the medulla of the thymus and the PLS of the spleen after stress

**Fig. 5:**
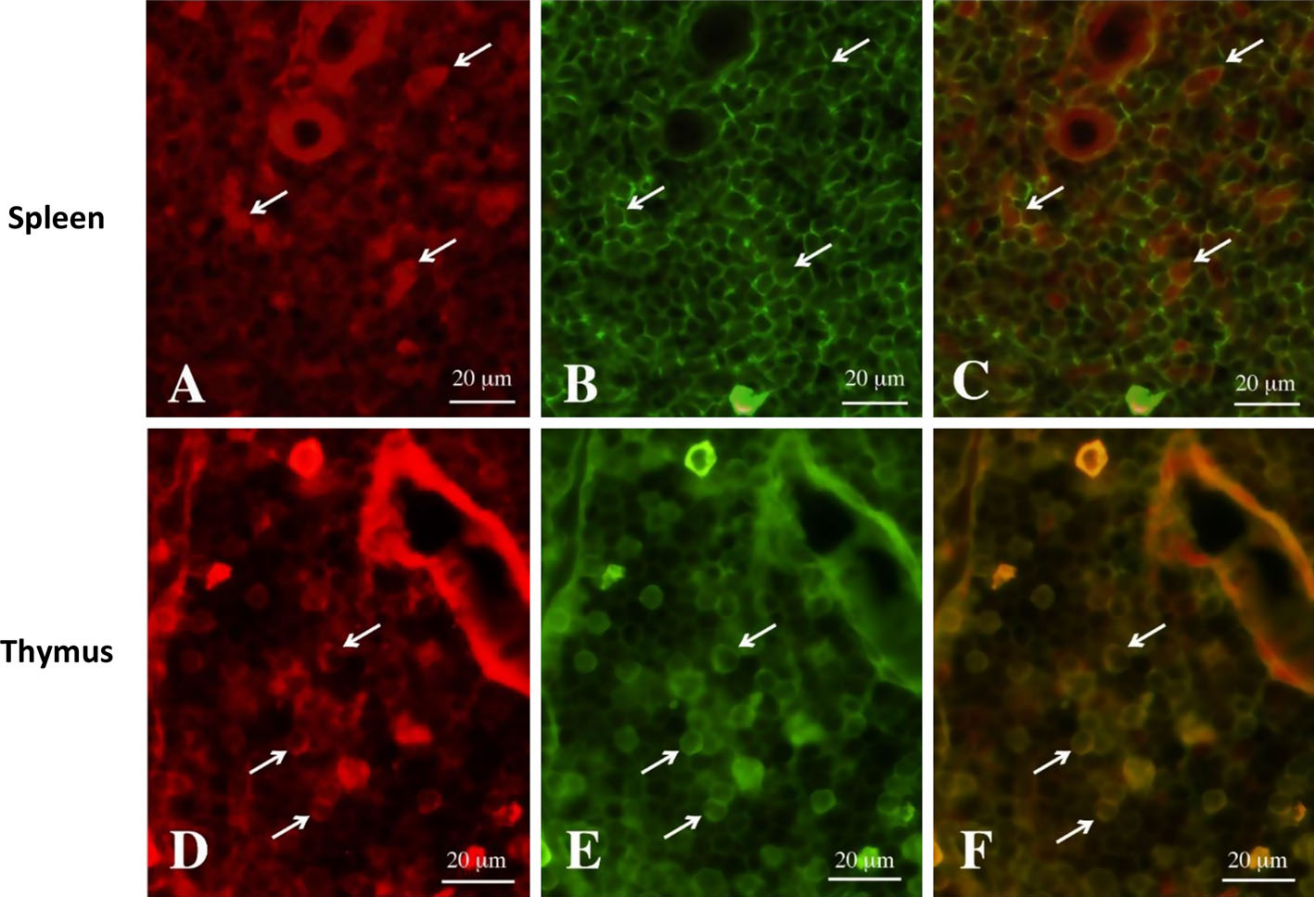
Localization of Gal-1 and CD45^+^ in the spleen and thymus with immunofluorescent microscopy Gal-1 immunoreactive (**a**, **d**) and CD45 immunoreactive (**b**, **e**) cells, and marged photographs (**c**, **f**) in identical sections of the spleen (**a**–**c**) and thymus (**d**–**f**) from 30 min survived animals after stress. *Arrows* in **a**–**c** and in **d**–**f** indicate identical cells showing Gal-1 immunoreactive and CD45 immunoreactive cells, respectively. *Scale bars* = 20 μm

We next determined the cell types that were Gal-1 immunoreactive in the SR30 spleen and thymus using fluorescence microscopy. The co-localization of Gal-1 and CD45, a marker of lymphocytes, was analyzed using double staining for Gal-1 and CD45 (Fig. 5). The results indicated that Gal-1 immunoreactive cells were immunopositive for CD45 in the medulla of the thymus and the PLS of the spleen.

## Discussion

Physical restraint stress has been widely used as a standard experimental procedure for studying physiological and/or psychological stress effects [19–22]. This type of stress induces pathological physiology of the endocrine system, autonomic nervous system, and immune system. Stress involves activation of the HPA axis and autonomic nervous system with consequent increases in plasma corticosterone and catecholamines [23]. Restraint stress induces circulating glucocorticoids (GCs), which immediately increase the percentage of lymphocytes undergoing apoptosis in immune organs via binding and activation of GC receptors [24, 25]. Our previous study revealed that the increase in stress-induced plasma Gal-1 levels is immediate and significantly higher than in the control [16]. Gal-1 induces apoptosis of activated T lymphocytes by binding several receptors such as CD7, CD43, and CD45 [14, 26]. Our current study suggested that stress-induced Gal-1 in serum after restraint stress accumulation in the thymic and splenic lymphocytes that were immune-positive for CD45 implying some interactions between Gal-1 and CD45.

Western-blot analysis demonstrated a significant increase in Gal-1 protein in the thymus and spleen in the SR30 and SR60 groups (Fig. 1b). Previous reports showed that Gal-1 is expressed in the thymus and spleen [26, 27]. The Gal-1 gene was expressed in both organs, and the level did not change after restraint stress as observed with RT–PCR (Fig. 2). These results suggested that stress-induced Gal-1 accumulated immediately in the spleen and thymus after stress, possibly to prevent physiological and/or psychological stress through the immune response.

On the other hand, in the immunohistochemical study, the immunostaining intensity of Gal-1 protein increased from the S group to the SR60 group in the medullary region in the thymus compared with the C group; this increase was not seen in the capsular and cortical regions. Immunoreactivity for Gal-1 was detected in the PLS region in the spleen in a similar time-dependent manner, but this increase was not seen in red pulp or the capsular region (Fig. 3). The results indicated that the Gal-1 immunopositive area showed regional specificity in both organs following restraint stress. The thymus is the primary lymphoid organ and contributes to the generation and maturation of lymphocytes. In particular, the medullary region in the thymus plays a key role in T cell tolerance and prevention of autoimmunity. The spleen is a secondary lymphoid organ that produces lymphocytes in the white pulp region and PLS, which contain T lymphocytes. Previous studies suggested that restraint stress immediately increases the percentage of lymphocytes in both organs and that the lymphocytes undergo apoptosis, which is mainly induced through GC receptors on lymphocytes following stress and the stress-induced increase in circulating GC [24, 25]. Interestingly, the present study demonstrated that Gal-1 immunoreactivity was present in the region of the immune response and immune tolerance in the spleen and thymus, respectively, and the immunoreactivity increased after restraint stress, although the Gal-1 gene expression level did not change.

Gal-1 induces apoptosis of activated T lymphocytes to maintain central and peripheral immune tolerance through binding of cell surface glycoproteins such as CD7, CD43, and CD45 [14, 23]. In particular, CD45 is expressed on the leukocyte cell surface including on lymphocytes, monocytes, and neutrophils but not on erythrocytes or platelets [28]. CD45 is an essential marker of lymphocytes. Furthermore, this receptor appears to be essential for triggering Gal-1-induced apoptosis [14]. Therefore, double immunofluorescence was performed to examine if CD45^+^ lymphocytes were co-localized with Gal-1 in various regions of each organ after stress. Gal-1 immunoreactivity was co-localized with CD45^+^ lymphocytes in both organs in the SR30 group. Perillo et al. [14] demonstrated that 10 μM Gal-1 is necessary to induce T cell apoptosis, and furthermore, exposure of T cells to Gal-1 induces apoptosis over a period of 30 min to 6 h in vitro. Stress-induced Gal-1 accumulated in lymphoid organs, at least in the spleen and thymus. Although the local concentration in the present study was not known, Gal-1 may affect immunological function in both organs for a long period after 30 min of stress. These results suggested that CD45^+^ lymphocyte apoptosis induced by modulation of stress-induced Gal-1 is a novel mechanism of immunological adaptation in lymphoid organs following restraint stress.

In conclusion, the present study suggested that Gal-1, which is increased in serum immediately after restraint stress, may trigger lymphocyte apoptosis in each lymphoid organ by binding to CD45^+^ lymphocytes. The spleen and thymus were the target organs of stress-induced Gal-1 in serum, at least in part. Stress-induced Gal-1 in serum modulated apoptosis in lymphoid organs, representing a novel mechanism for preventing physiological and/or psychological stress.

Further studies are required to examine the interaction and/or cooperation between stress-induced Gal-1 and GC in serum in several lymphoid organs regarding the apoptosis of lymphocytes following stressful situations, as well as the idea that stress-induce Gal-1 mediates apoptosis in CD45 antigen expression accompanied with the differentiation and maturation of leukocyte in lymphoid organs.
